# Survival analysis of 1148 women diagnosed with breast cancer in Southern Iran

**DOI:** 10.1186/1471-2407-9-168

**Published:** 2009-06-05

**Authors:** Abbas Rezaianzadeh, Janet Peacock, Daniel Reidpath, Abdolrasoul Talei, Seyed Vahid Hosseini, Davood Mehrabani

**Affiliations:** 1 Nemazee Hospital Cancer Registry Center, Shiraz University of Medical Sciences, Shiraz, Iran; 2 School of Health Sciences and Social Care, Brunel University, London, UK

## Abstract

**Background:**

While there has been much research regarding risk factors and prognostic factors for breast cancer in general, research specific to Iran is sparse. Further, the association between breast cancer survival and socio-demographic and pathologic factors has been widely studied but the majority of these studies are from developed countries. Southern Iran has a population of approximately 4 million. To date, no research has been performed to determine breast cancer survival and to explore the association between the survival and socio-demographic and pathologic factors in Southern Iran, where this study was conducted.

**Methods:**

The data were obtained from the cancer registry in Fars province, Southern Iran and included 1148 women diagnosed with breast cancer between 2000 and 2005. The association between survival, and sociodemographic and pathological factors, distant metastasis at diagnosis, and treatment options was investigated using Cox regression.

**Results:**

The majority of patients were diagnosed with an advanced tumour size. Five-year overall survival was 58% (95%CI; 53%–62%). Cox regression showed that family income (good vs poor: hazard ratio 0.46, 95%CI; 0.23–0.90) smoking (HR = 1.40, 95%CI; 1.07–1.86), metastases to bone (HR = 2.25, 95%CI; 1.43–3.52) and lung (HR = 3.21, 95%CI;1.70–6.05), tumour size (≤ 2 cm vs ≥ 5 cm: HR = 2.07, 95%CI;1.39–3.09) and grade (poorly vs well differentiated HR = 2.33, 95%CI; 1.52–3.37), lymph node ratio (0 vs 1: HR = 15.31, 95%CI; 8.89–26.33) and number of involved node (1 vs >15: HR = 14.98, 95%CI; 8.83–25.33) were significantly related to survival.

**Conclusion:**

This is the first study to evaluate breast cancer survival in Southern Iran and has used a wide range of explanatory factors, 44. The results demonstrate that survival is relatively poor and is associated with diagnosis with late stage disease. We hypothesise that this is due to low level of awareness, lack of screening programs and subsequent late access to treatment.

## Background

Breast cancer is the most commonly diagnosed malignancy among women in developed countries [1-3], and in some developing countries [4-6]. According to the report of the Iranian Centre for the Prevention and Control of Disease, Ministry of Health and Medical Education, 2000, Iran; breast cancer is the most prevalent cancer among Iranian women and accounts for 21.4% of all malignancies.

The prevalence of breast cancer in Europe and the USA is estimated between 8 to 10%. However, the lowest prevalence is seen in Asian countries, at about 1% [7]. In Iran the prevalence of breast cancer was reported as 6.7/1000 in 2002, which is even less than this [8]. While there has been substantial research published on risk factors and prognostic factors for breast cancer in general, research specific to Iran is sparse. Further, the association between breast cancer survival and socio-demographic and pathologic factors has been widely studied but the majority of these studies are from developed countries.

Iran has a total population of just over 70 million and almost all studies of breast cancer in Iran are from the capital, Tehran with a population of approximately 14 million. Most of these studies have not focused on survival and prognostic factors. To our knowledge, only two studies from Tehran have investigated breast cancer survival [9,10]. Southern Iran has a population of approximately 4 million and to date no study has determined breast cancer survival in this region or explored the relationships between the survival and socio-demographic and pathological factors. This paper presents the results of a study which fills this gap by determining five-year breast cancer survival for women with breast cancer in Southern Iran, and the impact of 44 explanatory factors.

Ethical approval for this study was obtained from the Research Ethics Committee of Shiraz University of Medical Sciences, Iran, and the Research Ethics Committee of the School of Health Sciences and Social Care of Brunel University, UK.

## Methods

This study used patients' records from Shiraz University Cancer Registry Centre, which is a hospital-based registry in a tertiary care centre which delivers oncology services to a population of approximately four million. This is the only centre which delivers oncology services in Southern Iran. Therefore, most probably all cancer patients come to this hospital for treatment. However a few of them may travel to other centers for treatment. The Cancer Registry was started on 1 January 2000 and so this study includes women who were diagnosed with breast cancer between 1 January 2000 and 31 Dec. 2005. During that period, of the 6253 patients diagnosed with ten most common cancers in the area, 1192 were registered as having female breast cancer. Thirty women were excluded due to previous breast cancer (23), ductal carcinoma in situ (2), and other previous cancers (5). In addition, 14 women with bilateral tumours were excluded due to the small numbers. Thus, the study population comprised 1148 women who were diagnosed with a first primary invasive breast malignancy and who underwent breast surgery including axillary dissection.

All patients were followed-up at regular three month intervals for the first year following diagnosis and had regular six month follow-ups thereafter. The last date of follow-up was 29^th ^July 2006. All subjects in this study underwent surgery and received radiation and all except a very small proportion received chemotherapy. These three treatment options have been offered in three sequences: surgery followed by chemotherapy and then by radiation, chemotherapy followed by surgery and then by radiation, and surgery followed only by radiation, in common with current practice in this region. About 82% received surgery followed by chemotherapy and then by radiation, 16% chemotherapy followed by surgery and then by radiation, and 2% received surgery followed only by radiation. At the time of diagnosis all patients were evaluated for metastasis to five distant sites: bone, liver, lung, brain, and ovary.

The main objective of this study was to investigate the impact of a wide range of factors on breast cancer survival. Therefore, the only outcome considered here is survival. All variables recorded at the cancer registry (44) were used in this study. The 44 explanatory variables divide naturally into three groups: socioeconomic or demographic, clinical/pathological factors, and distant metastases.

The association between each of the explanatory variables and outcome was assessed in turn using Cox's regression (unifactorial analysis).

Variables that were significantly associated with survival were considered firstly in each of the three conceptual groups: socioeconomic and demographic factors together, clinical/pathological factors, and distant metastases. Each model included all variables from the particular group that were statistically significant unifactorially, and then the variable that had the greatest regression estimate p-value was removed from the model. This process continued by remodeling and repeating removal of the next variable with the greatest p-value until all variables left in the model had p-value less than 0.05. A final model was fitted by combining all variables which were statistically significant in the three groups separately.

The proportional hazards assumption was examined at all stages in two ways: i) visually by inspecting graphs of the cumulative baseline functions against log survival time and ii) by a test based on the Schoenfeld residuals.

The results are presented as hazard ratios and 95% confidence intervals. All analysis was conducted using Stata v9.

## Results

### Impact of socio-demographic factors on survival

Of the 1148 patients included in the analysis 859 were alive at the end of follow-up, 269 had died, and 20 were lost to follow-up. Median follow-up time, from first pathological diagnosis until the time of death or the end of study, was 34 months. Mean age at diagnosis was 47 years (ranged from 19 to 86 years).

In unifactorial analysis of all socioeconomic and demographic factors, only family income and smoking were significantly associated with survival. (Table 1) These two factors together were entered into the first model and both remained significant. Compared to the patients with a low family income those with a higher family income were at 54% lower risk of death. (Table 2) Smokers were also at a 40% higher risk of death compared to non-smokers.

**Table 1:** Uni-factorial analyses and distribution of socio-demographic factors

Unifactorial analysis			Distribution of factors	
	Hazard ratio (95%CI)	p	Numbers	%

Area of residence			1148*	100
Affluent	1	0.179	213	19
Middle	1.12 (0.79–1.58)		590	51
Deprived	1.36 (0.95–1.94)		345	30

Family income			1143	99
Low & poor	1	0.031	426	37
Moderate	0.88 (0.69–1.13)		669	58
High	0.44 (0.22–0.87)		48	4

Occupation			1148	100
Housewife	1	0.853	898	78
Non-manual	0.92 (0.67–1.28)		199	17
Manual	1.07 (0.63–1.80)		51	4

Education			661	57
Illiterate	1	0.939	203	18
Primary	1.01 (0.65–1.58)		223	19
High school	1.15 (0.71–1.85)		148	13
University	1.00 (0.58–1.74)		87	7

Smoking			1145	100
Non-smoker	1	0.009	904	79
Smoker	1.44 (1.09–1.89)		241	21

Marital status			1147	100
Single	1	0.056	104	9
Married	0.64 (0.44–0.93)		936	82
Divorced & Widowed	0.77 (0.46–1.28)		107	9

Blood group			408	36
O	1	0.739	153	13
A	1.18 (0.71–1.95)		105	9
B	1.32 (0.80–2.19)		100	9
AB	1.08 (0.53–2.20)		45	4

BMI (Body Mass Index)			951	83
<25	1	0.784	509	44
25–30	1.10 (0.82–1.47)		317	28
>30	1.09 (0.72–1.66)		125	11

No. of children			1143	100
≤ 3	1	0.218	571	50
4–8	0.95 (0.74–1.22)		518	45
>8	1.87 (1.18–2.96)		54	5

Age at diagnosis			1118	97
≤ 35 years	1	0.499	172	15
36–49 years	0.96 (0.67–1.37)		519	45
50–64 years	1.02 (0.69–1.50)		342	30
≥ 65 years	1.35 (0.82–2.22)		85	7

Age at first pregnancy			122	11
≤ 18 years	1	0.081	55	5
19–25 years	0.30 (0.08–1.06)		47	4
>25 years	0.21 (0.03–1.66)		20	2

OCP use			255	22
≤ 3 years	1	0.869	80	7
>3 years	0.96 (0.59–1.57)		175	15

Ethnicity			1140	99
Fars	1	0.821	1006	88
Non-Fars	1.04 (0.73–1.48)		134	12

Religion			1147	100
Islam (Shia)	1	0.931	1031	90
Islam (Sunni)	0.93 (0.65–1.32)		110	9.5
Others	1.23 (0-.)		6	0.05

Menarche age			938	82
≤ 13 years	1	0.934	561	49
>13 years	0.99 (0.75–1.30)		377	33

History of BC in SDR			1148	100
No	1	0.758	996	87
Yes	0.94 (0.65–1.36)		152	13

History of BC in FDR			1148	100
No	1	0.22	881	77
Yes	0.83 (0.62–1.11)		267	23

Duration of breast feeding			205	18
≤ 3 years	1	0.175	78	7
4–6 years	2.03 (0.96–4.29)		68	6
>6 years	1.62 (0.71–3.69)		59	5

Abbreviations: OCP = Oral Contraceptive pill, BC = Breast Cancer, FDR = First Degree Relatives, SDR = Second Degree Relatives

* Total number available of 1148 subjects

**Table 2:** Multi-factorial analysis by three conceptual groups and final model

Variables from the other three multifactorial analyses				Result of final model	
	Hazard ratio (95%CI)	P	**	Hazard ratio (95%CI)	p

Family income					
Low & poor	1	0.039	99%		
Moderate	0.90 (0.70–1.15)				
Good	0.46 (0.23–0.90)				

Smoking					
Non-smoker	1	0.016	99%		
Smoker	1.40 (1.07–1.86)				

Metastasis to bone					
No	1	0.000	100%		
Yes	2.25 (1.43–3.52)				

Metastasis to lung					
No	1	0.000	100%		
Yes	3.21 (1.70–6.05)				

Tumour size					
≤ 2 cm	1	0.000	88%	1	0.000
>2 & <5 cm	1.43 (0.97–2.09)			1.43 (0.97–2.09)	
≥ 5 cm	2.07 (1.39–3.09)			2.07 (1.39–3.10)	

Tumour grade					
Well-differentiated	1	0.000	88%	1	0.000
Moderately-differentiated	1.30 (0.88–1.93)			1.30 (0.88–1.93)	
Poorly-differentiated	2.33 (1.52–3.37)			2.33 (1.52–3.57)	

No. of involved lymph nodes					
0	1	0.000	88%	1	0.000
1–5	2.96 (1.78–4.94)			2.96 (1.78–4.94)	
6–10	5.29 (3.11–9.01)			5.29 (3.11–9.01)	
11–15	8.29 (4.98–13.80)			8.29 (4.98–13.80)	
>15	14.96 (8.83–25.33)			14.96 (8.83–25.33)	

Lymph node ratio					
0	1	0.000	88%	1	0.000
> 0 &≤ .3	2.02(1.30–3.13)			2.03 (1.09–3.76)	
> .3 &≤ .6	4.84 (2.72–8.60)			4.84 (2.72–8.60)	
> .6 &< 1	9.30 (5.48–15.80)			9.30 (5.48–15.80)	
1	15.31 (8.89–26.33)			15.31 (8.90–26.34)	

** Proportion of subjects in multifactorial analysis

### Impact of distant metastases on survival

Metastases to liver, lung and bone were all significantly associated with poorer survival in the unifactorial analyses (Table 3). Metastases to bone and lung remained significant in the multifactorial model. Compared to the patients without distant metastasis those with lung metastasis had just over three times, and those with bone metastasis had just over twice the risk of death (Table 2).

**Table 3:** Uni-factorial analysis and distribution of distant metastases

Unifactorial analysis			Distribution of factors	
	Hazard ratio (95%CI)	P	numbers	%

Metastasis to bone			1148	100
No	1	0.001	1110	97
Yes	2.18 (1.39–3.41)		38	3

Metastasis to liver			1148	100
No	1	0.001	1134	99
Yes	2.80 (1.48–5.27)		14	1

Metastasis to lung			1148	100
No	1	0.001	1134	99
Yes	3.06 (1.62–5.76)		14	1

Metastasis to brain			1148	100
No	1	0.283	1142	99.5
Yes	2.14 (0.53–8.63)		6	0.5

### Impact of clinical/pathological factors on survival

Of 15 clinical/pathological factors, greater tumour size and higher grade, tumour calcification and necrosis, skin and nipple involvement, vascular and lymphatic invasion, higher number of excised and involved nodes, greater lymph node ratio (LNR), treatment, and type of surgery were significantly associated with poorer survival in the unifactorial analyses (Table 4). The factors that remained significant were tumour size, histological grade, number of involved nodes, and lymph node ratio. Patients with tumour size 5 cm and above had a two-fold increase in risk of death compared to the patients with tumour size 2 cm and less. Patients with poorly differentiated tumour grades had a doubling of risk of death compared to those with well differentiated tumour grades. There was a steady rise in risk with increased number of involved nodes ranging from a three-fold to a fifteen-fold increase in risk compared to node negative patients (Table 2). Hazard ratios increased steadily as the lymph node ratio increased with the hazard being greatest for patients with a ratio of one compared with zero.

**Table 4:** Uni-factorial analysis and distribution of clinico-pathological factors

Unifactorial analysis			Distribution of factors	
	Hazard ratio (95%CI)	p	numbers	%

Tumour side			1064	93
Right	1	0.562	565	49
Left	1.08 (0.84–1.39)		499	4

Tumour location			440	38
Lateral	1	0.258	322	28
Medial	0.75 (0.42–1.36)		68	6
Central	0.60 (0.30–1.97)		50	4

Tumour size			1055	92
≤ 2 cm	1	0.000	298	26
>2 & <5 cm	1.82 (1.30–2.70)		523	46
≥ 5 cm	3.04 (2.05–4.50)		234	20

Tumour grade			1059	92
Well-differentiated	1	0.000	250	22
Moderately-differentiated	1.89 (1.28–2.78)		637	55
Poorly-differentiated	4.53 (2.99–6.86)		172	15

Nuclear grade			140	12
Low	1	0.068	33	3
Intermediate	10.88 (1.00–80.75)		91	8
High	5.70 (0.59–54.90)		16	1

Co-morbidity			352	31
GI&Respiratory	1	0.433	37	3
Cardiovascular	0.92 (0.44–1.95)		122	11
Psycho.&Neurological	0.69 (0.28–1.57)		55	5
Gynaecologic	1.42 (0.61–3.29)		39	3
Endocrine&Metabolic	1.13 (0.55–2.33)		99	9

No. of stillbirths			1097	96
0	1	0.581	997	87
1–3	1.19 (0.78–1.83)		92	8
>3	1.48 (0.47–4.62)		8	1

No. of abortions			1106	96
0	1	0.525	819	71
1–3	0.85 (0.64–1.14)		269	23
>3	0.80 (0.30–2.15)		18	2

Histological type			1062	93
IDC	1	0.086	942	82
ILC	0.68 (0.31–1.55)		33	3
Med.C	0.35 (0.14–0.84)		60	5
MLDC	1.55 (0-.)		3	<1
Muc.C	0.65 (0.09–4.62)		6	<1
IPC	1.33 (0.42–4.15)		10	1
Met.C	3.31 (1.05–10.39)		8	1

No. of excised lymph nodes			1048	91
0	1	0.007	20	2
1–10	1.48 (0.46–4.74)		263	23
11–20	1.93 (0.61–6.09)		553	48
21–30	2.29 (0.713–7.37)		189	16
>30	4.67 (1.28–17.03)		23	2

Lymph node ratio			1027	89
0	1	0.000	331	29
>0 & ≤ .3	1.98 (1.06–3.66)		246	21
>.3 & ≤ .6	5.24 (2.96–9.29)		156	14
>.6 & < 1	11.26 (6.68–18.96)		185	16
1	18.87 (11.09–32.08)		109	9

No. of involved lymph nodes			1046	91
0	1	0.000	349	30
1–5	3.03 (1.82–5.04)		346	30
6–10	6.59 (3.89–11.15)		146	13
11–15	10.16 (6.13–16.84)		123	11
>15	18.78 (11.21–31.45)		82	7

Tumour calcification			1022	89
No	1	0.000	740	64
Yes	1.67 (1.28–2.19)		282	25

Tumour necrosis			317	28
No	1	0.017	157	14
Yes	1.82 (1.11–2.97)		160	14

Nipple involvement			995	87
No	1	0.000	887	77
Yes	1.84 (1.32–2.57)		108	10

Skin involvement			1013	88
No	1	0.000	946	82
Yes	2.06 (1.39–3.05)		67	6

Vascular invasion			1035	90
No	1	0.000	604	53
Yes	1.90 (1.47–2.46)		431	37

Lymphatic invasion			1006	88
No	1	0.000	419	36
Yes	3.73 (2.67–5.20)		587	52

Treatment			1134	99
S&C&R	1	0.000	927	81
C&S&R	1.86 (1.39–2.50)		178	16
S&R	1.27 (0.56–2.87)		29	2

Type of surgery			1065	93
MRM	1	0.000	649	57
RM	1.09 (0.64–1.86)		62	5
TM	1.60 (1.13–2.28)		99	9
L	0.58 (0.37–0.90)		164	14
Q	0.32 (0.14–0.73)		85	7
PM	2.66 (0.85–8.36)		6	1

Abbreviations: IDC = Invasive Ductal Carcinoma, ILC = Invasive Lobular Carcinoma, Med.C = Medullary Carcinoma, MLDC = Mixed Lobular Ductal Carcinoma, Muc.C = Mucinous Carcinoma, IPC = Invasive Papillary Carcinoma, Met.C = Metaplastic Carcinoma, S&C&R = Surgery and Chemotherapy and Radiotherapy, C&S&R = Chemotherapy and Surgery and Radiotherapy, S&R = Surgery and Radiotherapy, MRM = Modified Radical Mastectomy, RM = radical Mastectomy, TM = Total Mastectomy, L = Lumpectomy, Q = Quadrantectomy, PM = Partial Mastectomy

We modeled all statistically significant variables of the three previous models together to explore how the effect of socio-demographic variables might influence survival. This analysis showed that while the effect estimates for all variables were virtually unchanged, the socio-demographic variables and bone and lung metastases became non-significant. (Table 2) We interpret this as showing that the effects of the real and measurable effects of socio-demographic factors on survival were expressed in tumour characteristics.

Using the life-table method, five-year overall survival of this study population was 58% (95%CI; 53%–62%). The three-year overall survival was 76% (95%CI; 73%–79%).

There was no evidence that the proportional hazards assumption was violated in any of the analyses reported above.

## Discussion

This study has shown that of all socioeconomic factors only family income was associated with survival after adjustment for other factors. This is in agreement with other studies which obtained family income data by interviewing patients [11-13] as in this study. However, other studies which have obtained income data from a census have shown no association [2,14,15].

Of all demographic factors assessed, only smoking was related to breast cancer prognosis in this study, showing an adverse effect on survival which remained significant after adjustment for income. Two other studies in the UK and Sweden found a similar result [16,17]. It is perhaps surprising that income remains significant after adjusting for smoking. This may be a true effect or may be due to inadequate control for smoking using the binary data available – smoker/non-smoker. Exploratory analysis provided evidence that the effects of smoking and income on survival were mediated through adverse tumour characteristics.

The impact of young age at diagnosis on breast cancer survival has been long debated. This study found no evidence of a relationship between younger age at diagnosis and survival. Moreover age at diagnosis was not related to tumour characteristics. These findings accord with some studies [13,18,19] but not others [20,21] although in the latter studies the age categorization and settings were different.

BMI was not significantly related to survival in agreement with Carmichael's study in 2004 [22]. However four other studies reported a higher risk of death in patients with BMI > 30 compared to those with BMI < 25 [23-25]. The differences in findings for BMI in our study could be due to their late stage diagnosis for our study population.

This study found no evidence for a relationship between family history of breast cancer and survival and also observed that patients with positive and negative family history had similar tumour characteristics. This is consistent with the results of several other studies [21,26,27]. Our findings revealed that tumour size, histological grade, and lymph node status were associated with breast cancer survival after mutual adjustment. This result is consistent with some other studies [28,29]. We found that poorly differentiated tumours carried a higher risk of death compared to well-differentiated tumours. Patients with tumour size 5 cm and above had a higher risk of death than those with tumour size 2 cm and less.

In this study lymph node status was investigated in three different ways: number of involved nodes, lymph node ratio, and number of excised nodes. The number of involved nodes and lymph node ratio were the most powerful predictors of survival on multifactorial analysis. According to our findings not only did node positive patients have a poorer survival rate compared to node negatives, but also as the number of involved nodes increased the risk of death increased too. A similar trend has been reported elsewhere [30,31]. Lymph node ratio (LNR) was negatively correlated with survival in agreement with studies from Canada [32], Belgium [33,34] and the USA [35]. The number of excised lymph nodes was non-significant after adjustment for other pathological factors.

This study found no association between histological type and survival in common with other works [29,36]. We found no evidence for an effect of intra-mammary tumour location. However, three studies reported an adverse effect of medial location [37,38], and three others reported that central location was a negative predictor of survival [39,40] compared to other locations. These differences might be due to missing data in our study since data regarding tumour location was only available for about one-third of the women. Skin and nipple involvement, tumour calcification and necrosis, vascular and lymphatic invasion were negatively associated with survival, but these effects became non-significant after adjustment. We note that these factors were closely correlated with each other and also with tumour size and grade, and lymph node status which may explain our findings.

Patients who underwent surgery as the first treatment option had a better prognosis than those who were treated firstly by chemotherapy. It might be due to a larger tumour size; because, women with larger tumours or metastatic diseases at diagnosis were mostly treated with chemotherapy followed by surgery. Moreover, all patients with tumour size above 1 cm received chemotherapy, which is a standard practice at the institution of study. This practice may not be standard elsewhere and this difference in treatment may contribute to the relatively poor prognosis seen.

Most other studies have reported significant effects on survival of tumour size, histological grade, and lymph node status but for other pathological factors. Our findings differed a little which could be due to the adjustment we performed – other studies have tended to adjust for only a few pathological factors whereas in our study we included 13 pathological factors that were significantly associated with survival in unifactorial analysis.

Three- and five-year overall survival rates in southern Iran were found to be 76% and 58%, respectively. To our knowledge only two studies have previously reported 5-year overall breast cancer survival rates in Iran and these were 60% [9] and 62% [10]. These studies were conducted in Tehran. The 5-year overall survival rates in Iran compare with 46% in India [41], 64% in Oman [4], 65% in Greece [42], 71% in Germany [43], 78% in Belgium [41], 89% in the USA [44], and 84% in the UK [45] and show that Iran has considerably poorer survival than European countries and the United States.

There are several possible reasons for this. In Iran women's awareness of breast cancer is limited – Iranian women have little or no information regarding breast self examination and its effect on early detection and prognosis. A study of health staff in Tehran found that only 6 percent of them reported doing breast self examination on a regular basis [46] and a study in Middle Eastern Asian Islamic immigrant women in the USA reported that none did regular breast self-examination [47]. Although, Hackshaw, 2003, concluded that breast self-examination cannot improve survival after breast cancer, women who do it, are more aware of changes in their breast and seek care earlier if there is any problem [48].

There are strong cultural barriers which hinder Iranian women from consulting with a physician for sensitive female-specific health problems. Even highly educated women are reluctant to seek treatment for breast tumours. Further to this, access to cancer treatment units is slow, delaying diagnosis and there is no screening mammography. It seems probable that all of these factors increase the chances of delayed diagnosis and hence late stage disease which is the main difference between Iranian women and women in Western countries.

There are some limitations to this study. Data for some explanatory factors were missing and some were recorded in a wrong way that made them less useful. For example, for age at first pregnancy data were available for 122 patients and for OCP usage it was available for 255 patients. In relation to OCP usage it was recorded as usage of OCP for three years and less and for above three years. It was not clear whether the others had never used OCP or they had used it but they were not asked for any information. A proportion of subjects had received hormonal therapy, but no data regarding hormone receptor status and hormonal therapy were recorded in the registry and so this factor could not be investigated. Also the type of chemotherapy drugs, doses, and duration of chemotherapy was not recorded at the registry and was not analyzed in this study.

In relation to preexisting diseases, only one disease was recorded for each patient per GP visit and so there were no data on any other preexisting diseases. In addition, preexisting diseases were categorized into five categories: gastrointestinal and respiratory, cardiovascular, psychological and neurological, gynaecological, endocrine and metabolic. This categorization differs from the international classification of diseases, and other diseases such as musculoskeletal and skin diseases were not considered. Again it was not clear whether the patients did not have these diseases or they had but it was not recorded. All of these limitations have since been addressed for future data collection but cannot be remedied for the current study.

It is strength and a weakness that this is the first study based on cancer registry data in Shiraz University of Medical Sciences, where full data collection was begun in 2000. The strengths lie in the richness of the data with many potential predictor variables tested and the uniqueness of the findings for this population. The weakness is that only 5 years of data were available for analysis, giving a relatively small sample size of 1148 women. This therefore limits the statistical power of the study. With 1148 women and power 90%, significance level 5%, a hazard ratio of 1.4 can be detected. Therefore we acknowledge that this study has insufficient statistical power to detect effects which are smaller than this and so it is possible that smaller effects have been missed. In future years when more data have been gathered, power will be greater and smaller effects can be estimated with greater confidence. In addition, in future years, the follow-up period will be longer allowing survival to be estimated with greater precision and to allow the estimation of survival beyond the 5 years possible at this time.

## Conclusion

In conclusion, the results presented in this study demonstrate a relatively low five-year overall survival rate for women diagnosed with breast cancer in Iran. Following the analysis of 44 explanatory factors, the results presented in this study suggests that survival from breast cancer in southern Iran is affected by delayed diagnosis and therefore late stage disease. We hypothesize that this is due to low level of awareness, cultural barriers and slow access to treatment. Further research is needed in Iranian women to test these hypotheses and thus design appropriate interventions to ultimately improve survival.

## Competing interests

No authors have any competing interests. This works is a part of a PhD thesis in epidemiology by AR examined at Brunel University in August 2008. Shiraz University of Medical Sciences funded the PhD program but played no role in the academic work or in the decision to publish.

## Authors' contributions

AR conceived the study and performed all of the data collection, statistical analyses and wrote the first draft. JP participated in the study design and advised throughout on the statistical analyses and writing. DR contributed to the direction of the study, the interpretation of the data and the writing. AT and SVH are surgical oncologists involved in the treatment of the subjects. DM took part in the process of registration and data recording.

## Pre-publication history

The pre-publication history for this paper can be accessed here:

http://www.biomedcentral.com/1471-2407/9/168/prepub
